# Enhancing the multi-dimensional assessment of quality of life: introducing the WHOQOL-Combi

**DOI:** 10.1007/s11136-020-02661-9

**Published:** 2020-12-17

**Authors:** Suzanne M. Skevington, Christine Rowland, Maria Panagioti, Peter Bower, Christian Krägeloh

**Affiliations:** 1 International Hub for Quality of Life Research, Manchester Centre for Health Psychology, Division of Psychological Science and Mental Health, University of Manchester, Manchester, M13 9PL UK; 2 NIHR School for Primary Care Research, NIHR Greater Manchester Patient Safety Translational Research Centre, Manchester Academic Health Science Centre, University of Manchester, Manchester, M13 9PL UK; 3 NIHR School for Primary Care Research, Centre for Primary Care, Division of Population of Health, Health Services Research and Primary Care, Manchester Academic Health Science Centre, University of Manchester, Manchester, UK; 4 Centre for Person Centred Research, Auckland University of Technology, Auckland, New Zealand

**Keywords:** Quality of life, Health, Measure, WHOQOL-combi, Psychometric, Validity

## Abstract

**Introduction:**

We revisited the global concept of subjective quality of life (QoL) as assessed by the WHOQOL-BREF to investigate whether it could be elaborated into a conceptually more comprehensive instrument with good psychometric properties. Responding to a growing need for shorter QoL measures with broader social, spiritual and environmental contents, facets from WHOQOL international modules were examined for potential integration into the new WHOQOL-Combi.

**Method:**

Adults over 65 years, diagnosed with one or more chronic diseases (*n* = 2833), completed 41 WHOQOL items during the CLASSIC survey; each item represented a WHOQOL facet. This pool of specific QoL facets contained 24 from the WHOQOL-BREF (excluding general items), and 17 from recent international WHOQOL short-form modules, selected for their generic properties. Rasch modelling reduced the final item pool when assessing the WHOQOL-Combi’s conceptual structure. Comparisons are made with the WHOQOL-BREF.

**Results:**

Modelling confirmed the tenability of a 36-item solution scored as a five-domain profile, comprised of 24 WHOQOL-BREF facets and 12 new facets from modules. Social and psychological domains were strengthened by three facets, spiritual QoL by five, and physical QoL by one. The WHOQOL-Combi showed sound model fit, excellent internal consistency (α = .95), and scores discriminated between socio-demographic categories. Concurrent validity with the EQ-5D-5L was confirmed for physical and psychological domains. Performance was similar to the WHOQOL-BREF.

**Conclusion:**

The WHOQOL-Combi offers a contemporary, comprehensive, integrated, multi-dimensional subjective QoL instrument with enhanced evaluations of social, spiritual, psychological and physical QoL. Acceptable to older people, future research should evaluate younger age groups and other cultures.

## Introduction

The quality of life (QoL) construct contains multiple, distinctive, concrete and important components, so a comprehensive item set is required to adequately assess it [1]. The present research responds to a growing need for shorter generic instruments that can comprehensively assess social, spiritual and environmental dimensions, alongside physical and psychological. The World Health Organisation definition of subjective QoL states six important QoL domains: *‘An individual’s perception of his/her position in life, in the context of the culture and value systems in which s/he lives, and in relation to his/her goals expectations, standards and concerns. It is a broad-ranging concept, incorporating in a complex way the person’s physical health, psychological state, level of independence, social relationships, personal beliefs, and relationship to salient features of the environment*’ [2]. These domains represent broad, overarching concepts within which clusters of similar facets (components) of QoL are organised. Facets are *behaviours, states of being, capacities, or subjective perceptions of experiences* [2]. Originally, the WHOQOL Group considered around 2000 possible questions pooled from focus groups held in 15 cultures world-wide. Using a cross-cultural survey, they piloted 276 items covering 30 facets of QoL [3]. Psychometric performance identified 100 items in 25 facets and scored them as 6 domains in the WHOQOL-100. These represent the core international features of QoL in the WHOQOL instruments [4, 5]. Twenty-six items were later extracted, to create a shorter WHOQOL-BREF measure [5, 6].

Administering long questionnaires may not be pragmatic as the quality of response can degrade, and rate of missing data increases. Although empirical evidence is scarce, the maximum recommended size is 50 items [7, 8]. Nevertheless, longer questionnaires can assess a more complete concept of life quality, and prompt valued self-reflection. A wider range of measured topics may also permit less common but important health problems to be detected that require clinical intervention [e.g. 9, 10]. In contrast, short measures have technical problems such as reduced reliability and limited content validity [11], although Rasch modelling has recently advanced item selection and measurement quality [e.g. 12]. Measures designed to assess a highly multidimensional concept with intermediate length may avoid some pitfalls.

The present study revisits the suite of WHOQOL multi-dimensional, cross-cultural instruments that were designed for sick and well adults to self-report their QoL [13]. After developing the WHOQOL-100 and WHOQOL-BREF core measures, additional modules of facets designed for attachment to the core, were developed and standardised to improve instrument relevance to a particular disease or condition, and enhance acceptability (e.g. perceptions of HIV symptoms [14]). However facets like social inclusion that emerged in the HIV/AIDS module, might also be relevant to different conditions or even the general population. Likewise intimacy in the WHOQOL-Old [15] could be equally relevant to younger people. Where generalisation was possible, adding facets to the core concept could elaborate and enhance it. As modular facets were also derived from a common, mutually agreed WHOQOL concept and protocol, they could be readily integrated into the core. Their inclusion offers a fine-grained QoL analysis through greater comprehensiveness with a more nuanced interpretation of the concept.

When reviewing the WHOQOL contents across instruments we noticed that selecting generic facets in these context-specific modules could have benefits if more widely administered. First, some module facets may have increased in relevance in the 25 years since the core was developed. Second, despite good international performance [6], the WHOQOL-BREF social domain was consistently weakest [e.g. 16, 17], so this research offered a chance to improve precision by increasing the amount of information currently limited by three items. Third, this research provided an opportunity to re-examine the QoL structure of the WHOQOL construct. Models of successive instruments displayed variable numbers of domains. There were four domains in the WHOQOL-BREF after collapsing the WHOQOL-100 physical and independence domains into physical QoL, and subsuming a solitary spiritual component within psychological QoL. The WHOQOL-SRPB-BREF [18] had five domains, and the WHOQOL-100 and WHOQOL-SRPB [19] six. Although scoring decisions were justified by the best psychometric evidence, occasionally alternative models were similar. Mindful of theoretical guidance from the QoL definition, our modelling contributes to debates about what constitutes the key components of a comprehensive, international concept of QoL and its measures.

Internationally standardised WHOQOL modules offered a rich item pool to this investigation. While most modules assess specialist populations (e.g. HIV [14], disability [20], old-age [15]), the WHOQOL spiritual, religious and personal beliefs (SRPB) module expands an existing domain of the core concept, so confirming that a spiritual QoL is conceptually distinctive, elaborated as nine facets, and pertinent to diverse cultures [19]. Each WHOQOL short-form facet is usually represented by one of its long-form items, so WHOQOL-BREF items extracted from the WHOQOL-100 replicate its structure. These provided the foundation of a new, parsimonious instrument we proposed to build. The research aimed to investigate whether the generic WHOQOL core concept could be enhanced by adding generic facets from modules to create the WHOQOL-Combi, and provide a measure of intermediate length with greater multidimensionality. This is important as many WHOQOL dimensions are not captured by other popular generic short assessments.

## Methods

### Sampling and recruitment

Data were collected through the Comprehensive Longitudinal Assessment of Salford Integrated Care (CLASSIC) survey of health in older patients. Participants were 65 years or over, registered with a general practice in Salford, UK, and diagnosed with one or more long-term health conditions (*n* = 3686) [21, 22]. This socio-economically deprived population has some of the nation’s poorest health. WHOQOL-Combi and EQ-5D-5L questionnaires were administered during the third follow-up (2016), 18 months after baseline. The EQ-5D-5L was recently modified from the EQ-5D ‘gold standard’ measure [23].

### Procedures

International and national WHOQOL modules (see Table 1), were reviewed for generic facets. In a conceptual review of facet definitions we examined module manuals, WHOQOL publications and internal WHO documents. Item wording in long form modules and procedures for selecting module items were examined. The psychometric performance of each item, domain and module was scrutinised. Measures were excluded if: (i) no short form had been standardised (e.g. children’s QoL [24]); (ii) the module was standardised in one culture (e.g. UK WHOQOL-Pain [25]), not the WHO minimum of three from different Continents; (iii) ‘experts’ had proposed or endorsed new concepts and/or items, without user input (e.g. poverty [26]), so the requirements of patient/person-reported outcome measures (PROMs) were not satisfied [27].

**Table 1:** WHOQOL modules

International modules for specific diseases and conditions
HIV and AIDs: the WHOQOL-HIV [14]
WHOQOL-HIV-BREF short form [28]*
Spiritual, religious & personal beliefs: the WHOQOL-SRPB [19]
WHOQOL-SRPB-BREF: short form [18]*
Old Age (ages 60 +):
The WHOQOL-OLD and its short forms [15]*
Disability: the WHOQOL-DIS [20]
National modules from 10 international centres [29]
Autism [30]
Modules—one culture only
Chronic pain (UK) [25]
Developments for new modules
Poverty (Bangladesh, Ethiopia, Peru, Thailand) [26]
Children (age 5–8 and their parents) (Thailand) [24]
Adolescents (age 13–18) (UK) [31]

*Included modules

Several issues emerged during item selection that needed attention, for instance, apparent duplication of concepts in more than one module. Retention could increase completion burden. Similar facet themes included perceptions of the future in *fear of the future* (WHOQOL-HIV [14]), and *past, present and future activities* (WHOQOL-Old [15]). However recurring themes also signalled that this concept could be pertinent. As one item per facet was needed to maintain parity, both items above were pre-tested with CLASSIC data. Second, contents sometimes overlapped *within* modules, e.g. *autonomy* and *participation/isolation* (WHOQOL-Old [15]). As their psychometric performances did not indicate differences, both were pre-tested. Third, an *intimacy* facet in the WHOQOL-Old was similar to *sex life* in the core, so replacement or supplementation was tested. Fourth, a *political rights and freedoms* dimension had been proposed for an international poverty module [26], a Thai children’s measure by their parents [24], and as a New Zealand national item [32]. As *political rights* extend beyond health, this was not considered further, but a *freedom* facet in the WHOQOL-Old was retained.

WHOQOL measures assess QoL in the past 2 weeks. Each item is rated on one of several 5-point Likert response scales [33, 34]. A new item from a module is normally inserted into the core at the end of the relevant response scale block to speed completion. Socio-demographic details on gender, age, educational qualifications, living and work situation were documented.

### Analysis

Module facets had been designated to a domain during instrument development, and their position in the structure was reassessed. Item pool analysis for the preliminary WHOQOL-Combi was conducted without modular boundary constraints. Item reduction followed previous exclusion procedures [4, 5]. Negatively phrased items were reversed. Raw scores were calculated for items in the five provisional domains, then transformed linearly onto a 0–100 scale. Embedded WHOQOL-BREF items were scored as four domains.

Domain scores were not calculated if two or more items were missing. A two-item mean was calculated for the general facet. Cases were deleted where > 20% of items in the scale were missing. When no more than two items were missing, means were imputed from that person’s remaining domain items. During item reduction, item properties were tabulated: normality statistics, ceiling/floor effects and internal consistency reliability (ICR). When mapping the preliminary structure, Pearson Product Moment correlations (*r)* between items, domains and general QoL were calculated, and item-total correlations corrected as applicable; the acceptance cut-off was > .40 and significance *p* < .05 *(*one-tailed)*.* Where two or more results were unacceptable, item exclusion was considered. Where performance was mixed, items were retained for further psychometric testing.

The tenability of the hypothesised factor structure was investigated through confirmatory factor analysis (CFA) and Rasch analysis using non-imputed scores. Informed by the established factor structure of WHOQOL instruments/modules, a higher-order five-factor model (physical, psychological, social, environment and SRPB) was initially tested using CFA with diagonally weighted least-squares and polychoric correlations [35] (LISREL v.8.80 [36]). Since the overall sample size well exceeded recommendations for asymptotically distribution-free methods [35], analyses were conducted on split samples to demonstrate robustness via replication. Consequently, two samples of *n* = 1000 were randomly extracted from the overall sample: Sample A for the main CFA, and Sample B for replication. Following WHOQOL validation procedures [26], item error co-variances were not correlated, providing a conservative exploration of model fit.

Since Chi-square inflates with sample size [37] , model fit was assessed using the indices and root-mean-square error of approximation (*RMSEA*) < 0.06, comparative fit index (*CFI*) > 0.95, and standardized root-mean-square residual (*SRMR*) < 0.08 [38]. As these cut-offs may be uncertain for asymptotically distribution-free methods [38], the entire pattern of fit indices was interpreted. Following earlier research [32, 39], the hypothesised factor structure was further confirmed using Rasch analysis with an additional randomly selected *n* = 500 (Sample C), which met Rasch sample size criteria [40]. This was conducted in parallel, to resolve misfit sources in CFA models. This advantageous approach also provided confidence that the robustness of the factor solution could be generalised to a different psychometric method. Partial-credit Rasch analysis (RUMM 2030 software [41]) followed previous research [42] by using a domain-level subtest approach. This permits the differentiation of local dependency arising due to multidimensionality (trait dependency) from local dependency due to method effects (response dependency) [43]. If Chi-square fit for item-trait interaction is non-significant (*p* > .05, Bonferroni adjusted), and if Smith’s test confirms the uni-dimensionality of this domain-level subtest solution [44], then this higher-order WHOQOL structure is confirmed. Misfit was investigated with domain level analyses [32] before repeating subtests. Lastly, Differential Item Functioning (*DIF*) examined how much subtests perform differently by gender, age and living alone.

Discriminative validity was estimated by comparing ‘extreme’ groups for socio-demographic characteristics. Means, standard deviations (*SD*) and a significance test (*F*) were calculated for domains. We were unable to make health status comparisons between sick and healthy subgroups, as all participants were diagnosed with chronic conditions. Construct validity was established by examining concurrent validity and through Rasch modelling. Concurrent validity involved correlating WHOQOL-Combi domains with EQ-5D-5L domains [23].

## Results

### Sample

The sample contained 2833 participants, mean age 74.91 (SD 6.37), of which 46% were men and 52% women (2% missing from gender analysis). Thirty-four % lived alone, and 40% without a spouse/partner. Nineteen % had professional qualifications; 7% a university degree. Although 83% were retired from paid work, 5% received pay; the remainder did voluntary work, could not work, or were family carers. The majority identified as white British (94%), 3% as ‘other’ white, and 3% mixed ethnicity, Asian or Caribbean. Missing data was low and largely from social (4%) and spiritual (0.5%) domains.

### Preliminary features of the WHOQOL-Combi

Table 2 shows the 43 facet items tested for inclusion in the WHOQOL-Combi. A general WHOQOL-BREF facet (containing two items on overall QoL, and general health) was excluded from specific item analyses. The 41 specific facets were comprised of 24 WHOQOL-BREF items, and 17 items extracted from facets in three short-form modules: the WHOQOL-HIV-BREF [28], WHOQOL-Old [15] and WHOQOL-SRPB-BREF [18]. The preliminary WHOQOL-Combi was scored as: physical, psychological, social, environment and spiritual domains. As the WHOQOL-Old scored its modular items in an ‘Old’ domain [15], these were reallocated to a domain nominated during the pilot study, for reassessment. The position of 17 module items (*italics*) are shown in Table 2: *sensory functioning* (physical); *achievement, fear of the future, autonomy* and *freedom* (psychological); *social inclusion, use of time, intimacy,* and *blame* (social); none for environment, and eight new facets on spiritual, religious and personal beliefs (SRPB) with the single original WHOQOL-BREF spiritual item, totalling nine.

**Table 2:** The structure of the WHOQOL-Combi scored in 5 domains

**General overall quality of life and health Q1 & Q2**				
Domains				
Physical health (8 facets; 1 new)	Psychological (8 facets; 3 new)	Social relationships (6 facets; 3 new)	Environmental (8 facets; 0 new)	Spiritual, religious & personal beliefs (6 facets; 5 new)
Facets				
Pain and discomfort Q3 (R)	Positive feelings Q5	Personal relationships Q35	Physical safety and security Q14	Meaning in life$ Q12
Energy and fatigue Q17	Thinking, learning, memory and concentration Q13	Practical social support Q37	Home environment Q38	***Spiritual connection + Q9***
Sleep and rest Q31	Self-esteem Q34	Sex life Q36	Financial resources Q19	*Purpose in life* + *Q10*
Sensory functioning* Q30	Body image and appearance Q18	*Social inclusion % Q24*	Health and social care: availability and quality Q39	*Awe and wonder* + *Q25*
Mobility Q29	Negative feelings Q43 (R)	Use of time* Q41	Opportunities for new information and skills Q20	*Wholeness and integration* + *Q42*
Activities of daily living Q32	Achievement*# Q23	Intimacy* Q6	Opportunities for recreation and leisure Q21	***Spiritual strength + Q26***
Dependence on medication/treatment Q4 (R)	Fear of the future*% Q8 (R)	***Blame% Q7 (R)***	Physical environment Q15	*Inner peace and harmony* + *Q27*
Working capacity Q33	Freedom* Q16		Transport Q40	*Hope and optimism* + *Q28*
	Autonomy* Q22			***Faith + Q11***

(From a pool of 43 facets, the WHOQOL-Combi contains 38 items (in 37 facets) of which 12 are new)

**Bold**, facet deleted from final measure; *Q*, question number; R, reversed scoring;* italics*, new facet items extracted from the following modules: *, WHOQOL-OLD (*n* = 6); %, WHOQOL-HIV (*n* = 3); +, WHOQOL SRPB (*n* = 8); #, facet formerly named ‘Past, Present and Future Activities’ in WHOQOL-Old; $, original spiritual facet in WHOQOL-BREF

### Item reduction

Table 3 displays findings that guided preliminary item reduction for 41 items. Item means > 3.0 indicated good QoL. Most showed acceptable skew and kurtosis (< 1.00). Of the items with mild deviations from normality, a large *SD* was found for *spiritual connection;* and *blame* had elevated skewness. Ceiling and floor effects were identified where > 10% of the sample endorsed one extreme response option on the 5-point scale. The items *spiritual connection, spiritual strength, blame* and *faith* showed unacceptable floor effects; *social inclusion* had a ceiling effect.

**Table 3:** Item information used to select and reduce a pool of 41 specific facet items for the WHOQOL-Combi (*n* = 2774–2827)

WHOQOL-Combi facet items	Mean	Std. deviation	Skew	Kurtosis	Alpha with item deleted	% Very poor (1)	% Poor (2)	% Neither good nor poor (3)	% Good (4)	% Very good (5)
Pain and discomfort#*	3.56	1.16	− .38	− .80	.949					
Dependence on medicat’n/treatment#*	3.31	1.15	− .08	− .95	.950					
Positive feelings*	3.76	.79	− .88	1.24	.948					
*Intimacy*	3.66	1.03	− .77	.19	.949	5.8	12.1	21.7	43.8	16.7
***Blame#***	**4.40**	**.92**	**− 1.59**	**1.92**	**.950**	**63.6**	**21.8**	**8.6**	**4.8**	**1.3**
***Fear of the future#***	**3.93**	**1.03**	**− .81**	**.05**	**.949**	**35.6**	**35.4**	**18.0**	**8.4**	**2.6**
***Spiritual connection***	**1.97**	**1.27**	**1.02**	**− .30**	**.951**	**52.9**	**17.5**	**10.2**	**14.3**	**5.2**
*Purpose in life*	3.35	1.01	− .47	− .27	.948	6.0	15.6	29.7	39.2	9.5
***Faith***	**2.35**	**1.33**	**.55**	**− .99**	**.951**	**35.0**	**22.4**	**14.9**	**18.6**	**9.1**
Meaning in life*	3.49	.96	− .57	− .06	.948					
Cognitions*	3.62	.82	− .49	.34	.948					
Physical safety and security*	3.77	.78	− .61	.84	.948					
Physical environment*	3.69	.82	− .50	.51	.948					
*Freedom*	3.79	.84	− .85	1.17	.948	2.2	5.8	22.5	53.3	16.1
Energy and fatigue*	3.64	.98	− .67	.16	.947					
Body image*	3.95	.98	− .87	.34	.948					
Financial resources*	3.92	.97	− .74	.21	.949					
Information and skills*	3.79	.95	− .79	.51	.948					
Leisure and recreation*	3.46	1.19	− .50	− .63	.948					
*Autonomy*	3.38	1.09	− .42	− .56	.947	6.6	18.6	25.2	36.8	12.8
*Achievement*	3.38	1.06	− .37	− .49	.947	6.1	17.1	29.3	34.9	12.6
*Social inclusion*	4.23	0.77	− .95	.95	.948	0.3	3.8	10.4	44.0	41.4
*Awe and wonder*	3.68	1.08	− .61	− .32	.948	5.2	13.1	21.1	36.1	24.5
***Spiritual strength***	**2.39**	**1.36**	**.56**	**− .99**	**.951**	**35.7**	**21.1**	**15.8**	**16.6**	**10.9**
*Inner peace*	3.30	1.10	− .37	− .60	.948	7.3	17.8	25.9	35.7	13.2
*Hope and optimism*	3.57	1.01	− .54	− .12	.947	4.6	11.5	27.0	39.4	17.6
Mobility*	3.74	1.17	− .72	− .39	.948					
*Sensory functioning*	3.74	.97	− .67	.12	.949	6.6	18.6	25.2	36.8	20.3
Sleep and rest*	3.33	1.08	− .40	− .63	.949					
Activities of daily living*	3.73	.99	− .76	.15	.947					
Working capacity*	3.28	1.11	− .42	− .52	.948					
Self-esteem*	3.79	.91	− .70	.39	.947					
Personal relations*	4.13	.89	− 1.13	1.38	.948					
Sex life*	2.90	1.16	− .18	− .69	.949					
Social support*	3.96	.84	− .71	.64	.949					
Home environment*	4.38	.79	− 1.44	2.51	.949					
Health and social care*	4.18	.80	− .93	.92	.949					
Transport*	4.19	.84	− 1.13	1.53	.949					
*Use of Time*	3.88	.87	− .74	.61	.948	1.7	5.7	21.3	49.0	22.3
*Wholeness and integration*	3.75	.89	− .57	.47	.948	2.3	4.3	29.5	43.9	20.1
Negative feelings#*	3.98	.81	− .70	.57	.948					

High score denotes poorer QoL. Total Cronbach’s* α* for WHOQOL-Combi = .950

#, item reversed in scoring; *Italics*, WHOQOL module facet items; *, specific WHOQOL-BREF items; %, % across 5-point response scales showing ceiling or floor effects; **Bold**, deleted items

### Internal consistency reliability (ICR)

Table 3 shows Cronbach’s *α* when each item was removed. Where values matched or exceeded *alpha* for the full scale (.950), then the item risked being redundant and did not contribute to reliability [45]. Unacceptable items were: *dependence on medication or treatment, blame, spiritual connection, inner strength* and *faith.* For 24 WHOQOL-BREF specific items, ICR = .931*,* so both instruments showed excellent ICR.

### Correlating WHOQOL-Combi components

Corrected item-total correlations were largely acceptable (*r* > .40) for facet items except *spiritual connection, blame, faith* and *inner strength*. *Intimacy* (.49) and *sex life* (.47) had similar item-total correlations. A strong association between *achievement* and the psychological domain (.82) confirmed its position. Items correlated more highly with an alternative domain were: *freedom* (.57), *social inclusion* (.57) and *awe* (.53) with the environment domain, and *sensory functioning* (.47) and *use of time* (.70) with the psychological domain. *Fear of the future* correlated − .38 with social QoL and with its designated domain. No WHOQOL-BREF items were more strongly associated with an alternate domain.

Item correlations with *general* QoL ranged from .12 (*faith*) *to *.67 (*autonomy; achievement*). *Blame, spiritual connection, faith* and *inner strength* revealed spiritual domain weakness. Strong associations with the core were found for *autonomy, achievement, peace, hope* and *time use*.

In summary, 17 facet items were selected from three international short-form modules. Five facets showed two or more measurement problems, so were candidates for exclusion: *blame, fear of the future, spiritual connection, faith* and *spiritual strength*. Both *sex life* and *intimacy* were retained. Although four WHOQOL-BREF core items were weak, they had been previously endorsed by cross-cultural consensus [5, 6], so were retained. Departures from the global structure are expected for individual cultures, so these UK results do not threaten the integrity of the international measure.

### Construct validity

The CFA goodness-of-fit indices (Table 4) resulted from testing the baseline five-factor model (Model 1) based on known factor structures from previous instruments, with additional modular items assigned through conceptual similarity. Sample B results were slightly better than Sample A; the *CFI* > 0.950 criterion for Model 1 was met for Sample A, but *RMSEA* and *SRMR* still indicated substantial misfit in both. As modification indices and factor loading patterns did not reveal misfit sources, auxiliary Rasch results with Sample C were considered before continuing with CFA.

**Table 4:** Goodness-of-fit indices from CFA, to test the suitability of a five-factor baseline model (Model 1), using Samples A and B (*n* = 1000 each), when items 9, 11, and 26 had been discarded (Model 2), and after discarding two further items (7 and 8) (Model 3)

Model	Sample	*CFI*	*RMSEA* (90-% CI)	*SRMR*
1	A	0.936	0.093 (0.091; 0.095)	0.113
	B	0.953	0.093 (0.091; 0.095)	0.106
2	A	0.970	0.080 (0.078; 0.082)	0.067
	B	0.967	0.082 (0.080; 0.084)	0.070
3	A	0.971	0.081 (0.079; 0.083)	0.070
	B	0.969	0.083 (0.081; 0.085)	0.072

RMSEA values are shown with 90% confidence intervals (all indicators three decimal places)

Rasch analysis commenced with an initial 41-item model (excluding general items), and no proposed domain structure. As Model A was a significant misfit *(χ*^2^(205) = 1794.47, *p* < .001), the subsequent step tested the five-factor structure using a subtest approach. When Model B combined all relevant items into domain subtests adequate fit was still not achieved (*χ*^2^(25) = 71.50, *p* < .001). In domain 5, three items on *spiritual connection* (Q9), *faith* (Q11), and *spiritual strength* (Q26) stood out with significantly elevated fit residuals (> 10.00, when the acceptable criterion is* − *2.50 to 2.50). In the next iteration (Model C), these three spiritual items were discarded from the SRPB subtest. While overall fit was no longer significant (*χ*^2^(25) = 26.42, *p* > .05), there were substantial residual correlations. The final Model D, resolved residual correlations between domain 1 and 2 subtests by merging them into a further subtest. This strategy is consistent with previous WHOQOL-BREF research [42]. It achieved a non-significant fit (*χ*^2^(20) = 11.25, *p* > .05), evidencing an overall higher-order solution.

**Fig. 1:**
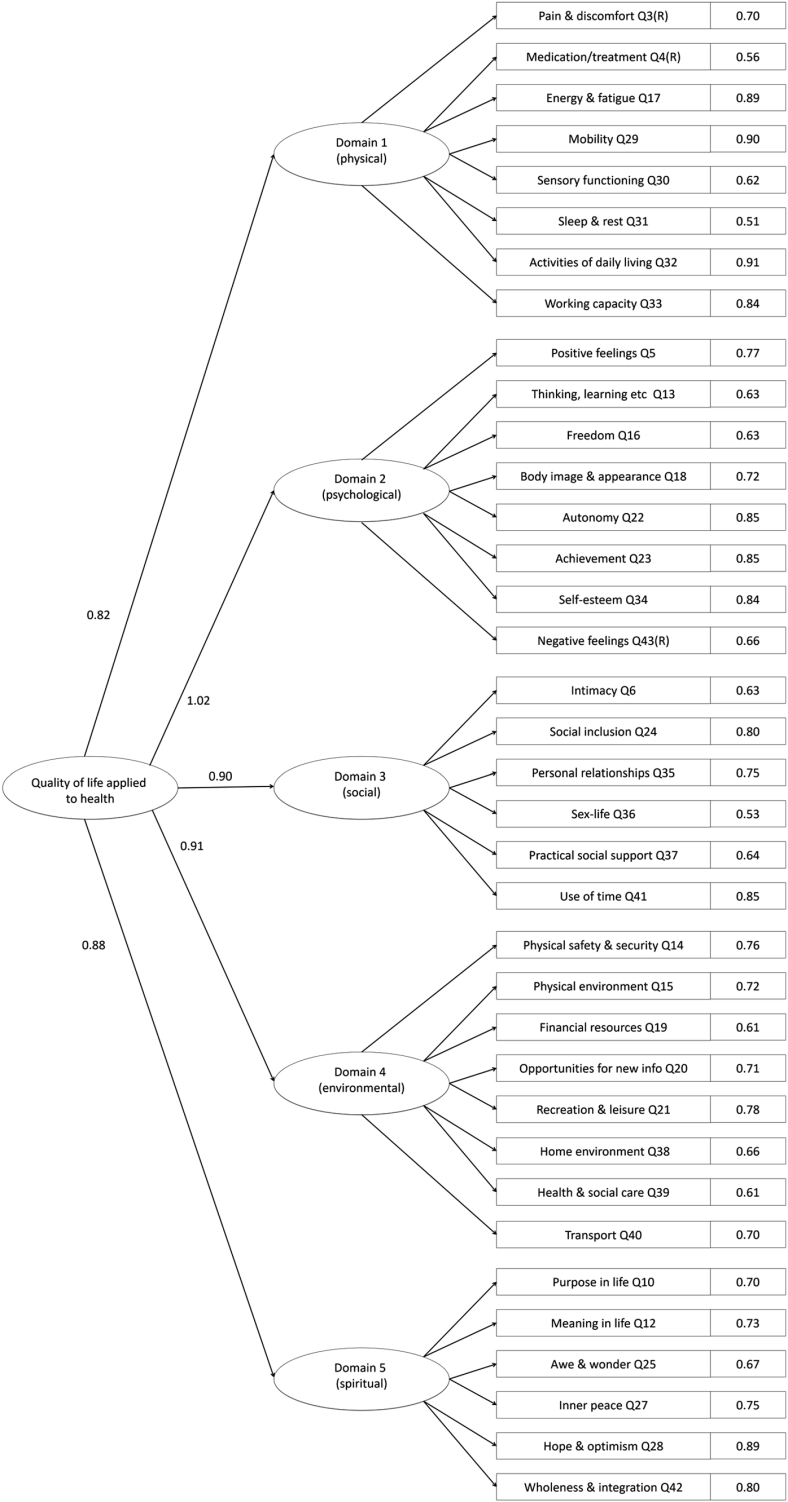
Results from fitting CFA Model 2 to the WHOQOL-Combi data from Sample A (*n* = 1000)

Model 2 (CFA) confirmed the tenability of the final model proposed after Rasch analysis where stand-out items (Q9, Q11, and Q26) had been removed from domain 5 (Table 4). Compared to Model 1, all fit indices substantially improved and now SRMR also met criteria of very good fit in both samples [41]. Although RMSEA still exceeded 0.060, such cut-off criteria may vary for asymptotically distribution-free methods, so it remains adequate in the context of other fit indices [41]. A marginal factor loading of 0.37 for *blame* (Q7), supported its deletion. Lastly, as *fear of the future* (Q8) showed a factor loading of 0.49, it was identified for removal to strengthen the measure. After deleting items 7 and 8, Model 3 continued to display excellent fit (Table 4; Fig. 1) and Rasch evidenced adequate unidimensional fit for this solution (*χ*^2^(20) = 11.36, *p* > .05), providing reassurance of robustness.

### Concurrent validity

WHOQOL-Combi domains correlated moderately with EQ-5D-5L domains [23] in Table 5. This set of results was stronger than for the WHOQOL-BREF. Physical and psychological domains showed strongest concurrent validity (*r* > .50). Compared with the WHOQOL-BREF, WHOQOL-Combi domains were strengthened by the inclusion of new facets. This improvement was especially important for the social domain, which had been weakest. As expected, the WHOQOL-Combi spiritual domain associated most weakly with the EQ-5D-5L domains, highlighting its unusual contribution to generic assessment.

**Table 5:** Pearson correlations between WHOQOL-Combi and WHOQOL-BREF domains with EQ-5D-5L domains/index (*n* = 2624–2776)

WHOQOL-Combi domains						WHOQOL-BREF domains			
EQ-5D-5L domains	Physical	Psych	Social	Envir’t	Spiritual	Physical	Psych	Social	Envir’t
Mobility (MO)	− .71	− .50	− .31	− .42	− .23	− .52	− .42	− .24	− .45
Self-care (SC)	− .59	− .47	− .30	− .38	− .24	− .46	− .42	− .22	− .39
Usual activities (UA)	− .74	− .57	− .36	− .46	− .28	− .57	− .47	− .26	− .47
Pain and discomfort (PD)	− .62	− .43	− .27	− .37	− .21	− .36	− .36	− .22	− .40
Anxiety and depression (AD)	− .47	− .63	− .49	− .45	− .35	− .43	− .49	− .39	− .49
Index (recoded)	.79	.64	.42	.52	.32	.51	.47	.29	.49

*WHOQOL Psych* psychological, *Envir’t* environment

All significant at *p* = .0001 (one-tailed)

### Features of the WHOQOL-Combi

Final WHOQOL-Combi inter-domain correlations (Table 6) were moderate to good. The spiritual domain was strengthened by removing three items. The social domain was now moderately correlated with other domains. WHOQOL-Combi domains correlated moderately strongly with *general* QoL. Correlation range is weaker for the WHOQOL-BREF in Table 6, possibly due to fewer items.

**Table 6:** Inter-domain correlations (transformed scores) for the total WHOQOL-Combi item pool, final WHOQOL-Combi items, and WHOQOL-BREF items (*n* = 2719–2832)

Quality of life domains	Physical	Psychological	Social	Environment
Total WHOQOL-Combi items				
Psychological	.76			
Social	.54	.74		
Environment	.66	.77	.70	
Spiritual	.42	.60	.58	.54
Final WHOQOL-Combi items				
Psychological	.75			
Social	.53	.74		
Environment	.66	.78	.70	
Spiritual	.58	.76	.72	.66
Embedded WHOQOL-BREF items				
Psychological	.63			
Social	.42	.54		
Environment	.60	.68	.56	

All correlations significant at *p* = *.*001

Table 7 presents means and *SDs* for key socio-demographic characteristics. When comparing QoL across age bands (60–69, 70–79, ≥ 80), all domains except social, significantly decreased over time. Environmental QoL declined only in over 80′s. Subsequent analyses therefore controlled age as a covariate (ANCOVA). Men reported higher physical and psychological QoL; women higher social QoL. Poorer QoL in all domains except physical, was associated with living alone. Holding a professional qualification was consistently linked to higher QoL. Retirees without paid work had lower physical QoL than paid workers.

**Table 7:** WHOQOL-Combi domain means (M) and standard deviations (SD), calculated as raw (range 4–20) and transformed scores (0–100), for socio-demographic characteristics

Domains	Physical		Psychological		Social		Environmental		Spiritual	
Variable	4–20 M (SD)	0–100 M (SD)	4–20 M (SD)	0–100 M (SD)	4–20 M (SD)	0–100 M (SD)	4–20 M (SD)	0–100 M (SD)	4–20 M (SD)	0–100 M (SD)
Men (*n* = 1300)	14.09 (3.17)	63.04 (19.79)	14.88 (2.77)	67.97 (17.30)	15.04 (2.72)	68.99 (17.00)	15.61 (2.47)	72.59 (15.42)	13.90 (3.27)	61.86 (20.45)
Women (*n* = 1481)	13.76 (3.22)	60.98 (20.12)	14.42 (2.78)	65.11 (17.39)	15.25 (2.69)	70.31 (16.83)	15.42 (2.53)	71.40 (15.80)	14.01 (3.22)	62.57 (20.15)
*p*	< .01**		< .01**		< .05*		< .05*		> .05	
Living alone (*n* = 963)	13.63 (3.19)	60.19 (19.93)	14.35 (2.92)	64.66 (18.25)	14.42 (2.94)	65.11 (18.35)	15.07 (2.65)	69.16 (16.57)	13.61 (3.47)	60.08 (21.68)
Not alone (*n* = 1814)	14.06 (3.20)	62.89 (19.98)	14.78 (2.70)	67.37 (16.88)	15.51 (2.51)	71.92 (15.71)	15.75 (2.39)	73.42 (14.92)	14.14 (3.12)	63.35 (19.47)
*p*	< .01**		< .01**		< .01**		< .01**		< .01**	
Profess- ional (*n* = 540)	14.47 (2.95)	65.47 (18.44)	15.02 (2.49)	68.88 (15.59)	15.47 (2.61)	71.71 (16.33)	16.14 (2.28)	75.90 (14.24)	14.60 (3.01)	66.26 (18.80)
Other (*n* = 2081)	13.81 (3.23)	61.30 (20.19)	14.57 (2.84)	66.04 (17.77)	15.06 (2.74)	69.11 (17.10)	15.40 (2.53)	71.24 (15.81)	13.83 (3.29)	61.44 (20.56)
*p*	< .01**		< .01**		< .01**		< .01**		< .01**	
Retired (*n* = 2352)	13.89 (3.13)	61.80 (19.59)	14.63 (2.74)	66.41 (17.14)	15.17 (2.68)	69.60 (16.73)	15.52 (2.47)	71.98 (15.43)	13.92 (3.21)	61.99 (20.09)
Other (*n* = 320)	14.49 (3.34)	65.56 (20.86)	14.90 (2.96)	68.14 (18.48)	15.45 (2.81)	71.59 (17.54)	15.75 (2.67)	73.42 (16.66)	14.34 (3.42)	64.66 (21.39)
*p*	< .01**		> .05		> .05		> .05		< .05*	
Age 60–69 (*n* = 799)	14.48 (3.20)	65.51 (20.03)	14.89 (2.78)	68.09 (17.38)	15.21 (2.77)	70.07 (17.30)	15.72 (2.56)	73.25 (16.00)	14.09 (3..19)	63.04 (19.97)
Age 70–79 (*n* = 1247)	13.97 (3.15)	62.34 (19.70)	14.70 (2.70)	66.88 (16.87)	15.21 (2.61)	70.06 (16.34)	15.78 (2.40)	72.37 (15.01)	14.12 (3.17)	63.28 (19.83)
Age ≥ 80 (*n* = 471)	12.98 (3.09)	56.14 (19.30)	14.18 (2.80)	63.60 (17.52)	15.01 (2.71)	68.80 (16.94)	15.15 (2.55)	69.71 (15.94)	13.51 (3.37)	59.47 (21.05)
*p*	< .01**		< .01**		> .05		< .01**		< .01**	

The *p*-value refers to significance from independent samples *t*-tests. As results were identical for scores in both range formats, only one *p*-value is shown per comparison

The WHOQOL-Combi SPSS syntax algorithm for scoring recodes five negatively worded items, calculates domain means from items, then multiplies by *4.00*. We recommend calculating means only where individuals complete a majority of items in each domain (maximum two items missing *per* domain). The profile of domain scores is transformed (0–100). Permission to use the WHOQOL-Combi and its scoring syntax, is obtainable from Dr Christine Rowland (christine.rowland@manchester.ac.uk).

## Discussion

We aimed to create a contemporary, fit-for-purpose measure based on the WHOQOL core concept that extended its conceptual scope. To do this, we selected quality, generic facets from three subsequent international WHOQOL modules to extend the concept of the established core. After examining the psychometric performance, we conclude that the new WHOQOL-Combi should contain 38 items, comprised of 12 drawn from 17 module facets, the 24 specific WHOQOL-BREF items and its two general items on overall QoL and health. Rasch modelling allocated the 36 specific items to one of five domains—physical, psychological, social, environmental or spiritual QoL—commensurate with the WHOQOL definition.

Preliminary psychometric properties indicated that the WHOQOL-Combi strengthen assessment performance by adding new facets to four domains. Simultaneously, it provided more equal numbers of items to the assessments; now six to eight items per domain, instead of three to eight. Doubling the social QoL facets from three to six, strengthened and elaborated this domain by adding *social inclusion, use of time* and *intimacy*. Although social remains the weakest domain [16, 17], the improvements suggest that the original three social facets were insufficient. Social QoL is important to assessing ageing populations where QoL can be undermined by chronic illness. The WHOQOL-Combi could be an asset to evaluating wellbeing interventions in old age [e.g. 22]. Psychological QoL was expanded by facets on *achievement, freedom* and *autonomy,* with potential to improve mental health assessment in many different applications e.g. pandemics. Although *sensory functioning* was added to physical QoL, new work should investigate its applicability to younger people. Adding a fifth, streamlined spiritual domain of six facets edited from nine in the WHOQOL-SRPB-BREF [18], is a notable feature of this shorter form.

While the 25 dimensions of the original core were arguably sufficient, three international modules contributed a further 12 distinctive QoL generic facets, so satisfying our purpose. As WHOQOL core and modular measures were developed from a common cross-cultural concept using culturally acceptable and feasible protocols, integrating new contents was straightforward. Psychometric modelling confirms that we succeeded in our aim. These findings cast light on the conceptual structure of the WHOQOL. Historically, WHOQOL models from successive instruments indicated that four, five or six domains should be scored, and this variation was puzzling. Although only the highest quality psychometric evidence had been prioritised when finalising instrument scoring, occasionally two models in the same series showed similar fit indices indicating that an alternative model was plausible. In the present study, the fit of two possible models was examined before consolidating a third solution, commensurate with common modelling practice.

Our investigation showed that CFA and Rasch models had a good fit, as sub-samples randomly selected for testing established five WHOQOL-Combi domains. This represents a conceptual departure from the four WHOQOL-BREF domains where the spiritual component was minimal, and neither distinctive nor strong. A new generic measure of intermediate length that includes spiritual QoL assessment is therefore an advance of the present work. Six spiritual facet items strengthen this WHOQOL-Combi domain, so replacing a solitary WHOQOL-BREF spiritual item located on the cusp of the psychological domain. WHOQOL-Combi scoring reaffirmed the five domain structure of the WHOQOL-SRPB-BREF [18]. Without elaborating SRPB, earlier WHOQOL instruments were conceptually incomplete, and this may account for scoring diversity. A substantial evaluation of spiritual QoL within a shorter generic assessment will permit its relatively obscure role in health outcomes to be elucidated in many populations.

Validity evidence showed that the generic WHOQOL-Combi and EQ-5D-5L measures were similar in the way they assessed physical and psychological QoL. As previous WHOQOL studies often incorporated the SF-36 for concurrent validity testing [17], this comparison with the recent EQ-5D-5L provides original findings. Furthermore WHOQOL-Combi scores could distinguish between key sociodemographic categories, so supplying useful validity information to applications.

The WHOQOL-Combi is a PROM based on international information that offers a more comprehensive facet profile than before, so could assist clinical decision-making in many conditions. As all participants had a diagnosed chronic disease, and little data was missing, this new measure seems acceptable to older people. Its 38 items in English can be completed in 10 min; more rapidly than the WHOQOL-100, where facet depth is an advantage. Although longer than the WHOQOL-BREF, this intermediate form shows good measurement properties, and does not exceed 50 items [7, 8]. Furthermore, this integrated instrument includes blocks of items that share the same response scale, so should be faster to complete than a battery of independent measures containing the same number of items. Technical and philosophical points on measurement choice deserve wider debate among healthcare professionals, who characteristically view length as the most important heuristic.

The CLASSIC survey of older adults with long-term illnesses provided sound cross-sectional data to standardise the new WHOQOL-Combi. As with the WHOQOL-BREF [46], intervention evaluation will improve after longitudinal data is available to test the sensitivity and responsiveness of scores to changing clinical and social conditions. Generalising to younger populations is not yet possible. As life expectancy increases, more oldest-old research will be needed. We studied a socio-economically deprived population with some of the poorest health in UK [22], so national surveys are feasible.

## Conclusion

The WHOQOL-Combi advances QoL measurement by drawing information from a suite of WHOQOL measures derived from a common international concept. Validated in UK, a cross-cultural survey should now examine this measure globally. It has innumerable policy uses, such as evaluating whether the Sustainable Development Goals improve QoL. With over 100 WHOQOL-BREF language versions available, adding the new module facet items after local cultural adaptation and translation, will speed access to the WHOQOL-Combi measure. As British-English versions of WHOQOL measures are the international reference point for other languages [13], this study adds value. As with the WHOQOL-BREF [10], detail in the WHOQOL-Combi profile can pinpoint QoL issues pertinent to patient-professional decision-making about care.
